# Relationship of maternal ophthalmic artery Doppler with uterine artery Doppler, hemodynamic indices and gestational age: prospective MATERA study

**DOI:** 10.1002/uog.29162

**Published:** 2025-01-20

**Authors:** S. Ali, D. C. Mukasa, D. Lukakamwa, A. Nakayenga, P. Namagero, J. Biira, J. Byamugisha, A. T. Papageorghiou

**Affiliations:** ^1^ Department of Obstetrics and Gynecology Makerere University Hospital, Makerere University Kampala Uganda; ^2^ Julius Global Health, Department of Global Public Health and Bioethics, Julius Center for Health Sciences and Primary Care University Medical Center Utrecht, Utrecht University Utrecht The Netherlands; ^3^ Department of Obstetrics and Gynecology Kawempe National Referral Hospital Kampala Uganda; ^4^ Nuffield Department of Women's and Reproductive Health University of Oxford Oxford UK; ^5^ Oxford Maternal and Perinatal Health Institute, Green Templeton College University of Oxford Oxford UK

**Keywords:** ophthalmic artery, peak systolic velocity ratio, pre‐eclampsia, pulsatility index, reproducibility, uterine artery

## Abstract

**Objectives:**

To examine the relationship of ophthalmic artery (OA) Doppler indices with uterine artery (UtA) Doppler indices, selected maternal hemodynamic parameters and gestational age, and to evaluate the intraobserver reproducibility of OA Doppler indices.

**Methods:**

This was a prospective cohort study of women recruited between 11 + 0 and 23 + 6 weeks' gestation using a stratified and random sampling approach to ensure adequate distribution across the gestational‐age range. OA pulsatility index (PI), first peak systolic velocity (PSV1), second peak systolic velocity (PSV2) and peak systolic velocity ratio (PSV ratio), calculated as PSV2/PSV1, were measured twice in each eye by the same observer. UtA‐PI was also measured twice on each side by the same observer. Maternal hemodynamic assessment was undertaken using an ultrasonic cardiac output monitor (USCOM 1A). Pearson's and Spearman's rank correlation coefficients were used to assess the correlations between variables, and Bland–Altman plots were used to evaluate the intraobserver reproducibility of OA Doppler indices.

**Results:**

Of 194 women invited to participate in the study, 169 were eligible for inclusion, of whom 16 were excluded following an obstetric ultrasound scan and a further three owing to inadequate or incomplete OA or UtA Doppler assessment, leaving 150 women in the final analysis. Log UtA‐PI had a weak correlation with both OA‐PI (*r* = –0.19 (95% CI, –0.34 to –0.03), *P* = 0.021) and OA‐PSV ratio (*r* = 0.31 (95% CI, 0.15–0.45), *P* < 0.001). The correlation between gestational age and OA‐PI was non‐significant (*r* = 0.14 (95% CI, –0.03 to 0.29), *P* = 0.097), and that between gestational age and OA‐PSV ratio was weak (*r* = –0.23 (95% CI, –0.38 to –0.07), *P* = 0.004), as opposed to the strong correlation between gestational age and UtA‐PI (*r* = –0.68 (95% CI, –0.76 to –0.58), *P* < 0.001). No strong correlations were observed between OA‐PI or OA‐PSV ratio and maternal hemodynamic indices. The correlations were unaltered by adjustment for maternal age and body mass index. The intraobserver reproducibility of OA‐PI and OA‐PSV ratio in the same eye was high. The correlation between the right and left eyes was moderate for OA‐PI (*r* = 0.63 (95% CI, 0.53–0.72), *P* < 0.001) and strong for OA‐PSV ratio (*r* = 0.81 (95% CI, 0.75–0.86), *P* < 0.001).

**Conclusions:**

OA‐PI and OA‐PSV ratio had a weak or no correlation with UtA‐PI and maternal hemodynamic parameters, meaning that they can be used as independent predictors for pre‐eclampsia. Gestational age had no clinically relevant effect on OA‐PI and OA‐PSV ratio, suggesting that these indices could be measured without adjustment at any time between 11 and 23 weeks' gestation. OA Doppler indices had high intraobserver reproducibility and were strongly correlated between the right and left eyes. © 2025 The Author(s). *Ultrasound in Obstetrics & Gynecology* published by John Wiley & Sons Ltd on behalf of International Society of Ultrasound in Obstetrics and Gynecology.

## INTRODUCTION

Pre‐eclampsia complicates 2–5% of pregnancies globally^1^, and low‐ and middle‐income countries (LMICs) bear a disproportionate share of this burden^2^. Efforts to reduce the rate of pre‐eclampsia entail the pairing of accurate prediction and prevention with aspirin^3^. Initially, prediction models were based on checklists of maternal and pregnancy history. By adding maternal uterine artery (UtA) Doppler indices, mean arterial pressure (MAP) and serum biomarkers, such as placental growth factor, valuable improvements in detection have been achieved. Although serum biomarkers improve diagnostic accuracy^4^, ^5^, they have not been implemented in most LMICs owing to the high cost of the tests and an often inadequate laboratory infrastructure^6^.

In the absence of liquid biomarkers, ophthalmic artery (OA) Doppler is a promising alternative for improving the prediction of pre‐eclampsia. Research has shown that OA Doppler can be used as a complementary tool with good diagnostic accuracy for pre‐eclampsia^7^, ^8^. Many studies have evaluated OA Doppler screening at 19–23 and 35–37 weeks' gestation^9^, ^10^, ^11^, ^12^, and recent research into its predictive ability at 11–13 weeks shows promise^13^, ^14^. However, data from sub‐Saharan Africa are sparse; studies from this region are small scale and are based on women with established pre‐eclampsia (and controls), rather than using OA Doppler in a predictive capacity^15^. In addition, previous studies have reported inconsistent findings on the relationship between OA Doppler and gestational age; some cite a significant correlation^16^, ^17^, while others dispute this finding^18^.

Most studies in high‐income countries examine models of pre‐eclampsia screening at 11–13 weeks. While in an ideal scenario, all women would be recruited to a study early in gestation, such a strategy is unlikely to be successful in settings where women seek care later in pregnancy. For instance, only 37% of women attend antenatal care during the first trimester in Uganda^19^. The average gestational age at first presentation in LMICs is > 20 weeks^20^. A screening program at 11–23 weeks is likely to be more suitable in settings where early antenatal care is lacking, and could widen access to clinical risk stratification, personalized follow‐up of women at higher risk and targeted use of prophylactic therapy, such as aspirin. Therefore, the aims of this study, conducted at 11 + 0 to 23 + 6 weeks, were to examine the correlation of OA Doppler indices with UtA Doppler indices, maternal hemodynamic parameters and gestational age, and to assess the intraobserver reproducibility of OA Doppler.

## METHODS

### Design and setting

The Maternal Antenatal Testing for Equitable Risk Assessment (MATERA) project aims to identify screening and prevention methods for major causes of maternal and perinatal morbidity and mortality in high‐burden settings. In this prospective cohort study, our primary aim was to assess the relationship between Doppler indices of the maternal UtA and OA between 11 + 0 and 23 + 6 weeks' gestation. This gestational‐age range was chosen after considering the potential for selection bias due to low attendance for early antenatal care and the importance of including higher‐risk women who typically present later in pregnancy. This compromise balances the ideal early screening period with real‐world considerations, ensuring broad inclusion and maximizing the potential benefit of clinical risk stratification and prophylactic therapy. Secondary aims were to assess the relationship between OA Doppler indices and selected maternal hemodynamic parameters, and to determine the intraobserver reproducibility of OA Doppler. This study was undertaken at Kawempe National Referral Hospital, a 200‐bed women's and children's facility in Kampala, Uganda, and a teaching site for Makerere University. The study was approved by the Research Ethics Committee (registration number: MH/REC/15/02‐2023).

### Participants

Pregnant women attending for routine antenatal care were approached to participate in the study by the midwife in charge, who was also trained as a research assistant. Participants were approached sequentially up to a daily quota for logistical reasons, while ensuring adequate distribution of gestational ages throughout the study. Women were eligible for participation irrespective of their risk profile for adverse pregnancy and neonatal outcome. A cross‐sectional approach was taken, with each woman contributing a single set of measurements between 11 + 0 and 23 + 6 weeks. As there is no reliable, routine clinical ultrasound service available for all women in this setting, an initial assessment to ascertain eligibility was undertaken for women considered to be < 24 weeks' gestation based on their last normal menstrual period or on information from any prior scans or an obstetric abdominal examination. Gestational age was then confirmed on ultrasound in all eligible women. Inclusion criteria were women aged at least 13 years who conceived naturally and provided consent to participate. Women with a gestational age > 23 + 6 weeks on the dating ultrasound scan, those with a fetal anomaly, multiple pregnancy or early pregnancy complications (e.g. miscarriage, ectopic pregnancy) and those found to be critically unwell and requiring hospital admission were excluded.

The women were counseled regarding the examinations they would undergo if they participated in the study, which included: an interviewer‐administered structured questionnaire that would elicit information on maternal factors; ultrasound examination and Doppler assessment; and maternal hemodynamic assessment. Measures were taken to ensure privacy; for example, all potential participants were taken to a separate room within the premises of the antenatal clinic to discuss participation in this research project. Pictorial illustrations of all elements of the study were shown to the women during the consenting process to improve understanding and mitigate fears about study procedures. After detailed explanation, comprehension was assessed by asking the women to explain the procedures back to the recruiting staff. Following this, written informed consent was obtained from all participants. All eligible participants were supported to access their routine antenatal care before undergoing study procedures. In addition, all participants underwent a free‐of‐charge obstetric ultrasound scan to inform their routine antenatal care. All measurements and procedures were carried out by qualified research staff who had undergone specific training and were supported by ongoing quality control.

### Maternal data

Participants underwent an interviewer‐administered structured questionnaire concerning their demographic characteristics and risk factors for pre‐eclampsia, including relevant obstetric and gynecological history, medical history and social background. Data were captured in an online case report form on KoboToolbox (Kobo Inc., Cambridge, MA, USA). Participants spent approximately 10 min responding to this questionnaire.

### Obstetric ultrasound

Following enrolment, participants underwent a transabdominal obstetric ultrasound scan, which was used to inform routine antenatal care and was reported to the managing clinicians. Ultrasound assessment was conducted using a Voluson E8 ultrasound machine (GE Healthcare, Zipf, Austria), equipped with C1‐6‐D convex and 9L‐D linear array probes. The first step was to confirm fetal viability, assess for multiple pregnancy and exclude major fetal abnormalities. Gestational age was estimated using fetal crown–rump length at 11–13 weeks of gestation^21^, or using fetal head circumference in combination with femur length beyond 13 weeks^20^, ^22^. This scan also included documentation of amniotic fluid level and placental location^17^. Participants deemed to be ineligible based on ultrasound findings were excluded at this stage.

### Doppler assessment

Doppler measurements were obtained using the same equipment as that used for the obstetric ultrasound scan. However, the results of UtA and OA Doppler assessment were not disclosed to the managing clinicians.

UtA pulsatility index (PI) measurements were obtained in duplicate from the right and left sides by the same observer, and the averaged values were used for analysis. With the woman lying in a semirecumbent position, transabdominal ultrasound with color Doppler imaging was used to identify the UtAs. This was performed using the recommended method specific to the gestational age: by obtaining a longitudinal cross‐section of the cervix and then moving laterally at 11–14 weeks^23^, or at the apparent crossover with the internal iliac artery thereafter^24^, ^25^. Measurements were obtained at a point along the UtA before it branches, with the zoom box and sample gate in the center of the UtA under assessment and the insonation angle < 30°, and at least three similar and consistent waveforms were obtained. Pulse‐rate frequency and color‐gain correction ensured image clarity, and the velocity scale was maintained at ≥ 75% of the peak velocity.

OA Doppler was evaluated in duplicate from the right and left eyes by the same observer. The first pair of measurements were used in the primary analysis, and the second set of measurements were used for the analysis of intraobserver reproducibility. With the woman in a supine position and the eye closed, sonogel was applied over the upper eyelid and a 9L‐D linear probe (GE Healthcare) was placed gently over the eye in a transverse orientation. The OA was identified using color‐flow Doppler imaging, superomedial to the hypoechogenic band representing the optic nerve. A minimum of three similar waveforms were recorded using pulsed‐wave Doppler, with an angle of insonation of < 20° (angle correction was used where necessary), sample gate of 2 mm, wall filter of 60 Hz and pulse‐repetition frequency of 2.0–4.0 kHz. As with any Doppler examination, the ALARA principle was adhered to, using the lowest possible output power and mechanical indices. OA‐PI, first peak systolic velocity (PSV1) and second peak systolic velocity (PSV2) were recorded, and the peak systolic velocity ratio (PSV ratio) was calculated as PSV2/PSV1.

### Maternal hemodynamic measurements

Maternal height (cm) and weight (kg) were measured using standardized scales. After an appropriate rest time of at least 5 min and with the participant in a seated position, an arm cuff was applied for the non‐invasive determination of blood pressure and MAP.

To gather data on maternal hemodynamics, assessment was undertaken using the USCOM 1A ultrasonic cardiac output monitor (Uscom Ltd, Sydney, Australia). This examination was performed with the woman in a left lateral position and the probes placed in the relevant positions with reference to the sternum, as per standard methodology^26^. With the probe in the suprasternal notch along the longitudinal axis of the sternum and ascending aorta, data on maternal hemodynamic function were obtained. The USCOM 1A device measures many hemodynamic indices; to avoid the risk of spurious correlations due to multiple testing, we made the *a‐priori* decision (based on a literature review) to examine MAP, heart rate, systemic vascular resistance (SVR), cardiac output, stroke volume and potential energy to kinetic energy ratio (PE/KE ratio).

### Data management

Data were entered in real time into electronic digital case‐report forms hosted on electronic tablets by research assistants. The study site had internet access, so the database was promptly accessible to the quality control manager (A.N.), study manager (D.C.M.) and principal investigator (S.A.). Every entry was reviewed by the quality control manager and study manager at the close of each day of data collection, ensuring near real‐time quality assurance and allowing for any missing data to be collected promptly. Other members of the study team had no access to the online database.

### Statistical analysis

Statistical analysis was performed using R version 4.3.1 (R Foundation for Statistical Computing, Vienna, Austria). The required sample size was calculated using the R package ‘pwr’, in addition to the formula for sample‐size estimation when the objective is to establish correlations between measurements, and using data from previous studies reporting a significant positive correlation between UtA Doppler and SVR in high‐income settings^27^, ^28^ (owing to limited data from low‐resource settings). At a 5% significance level for a two‐tailed test, with 90% power to detect a significant correlation between the measurements and with a correlation coefficient of 0.26, a sample of 150 women was required. Categorical data were presented as *n* (%) and continuous variables were presented as mean ± SD or median (interquartile range (IQR)), as appropriate. Non‐normality was determined using visual assessment (density distributions and Q–Q plots) and the Shapiro–Wilk test. Measurements > 5 SD away from the mean were defined as outliers.

The primary analysis of the relationship of OA Doppler indices with both UtA Doppler indices and maternal hemodynamic parameters used the first set of right and left OA measurements. As part of a preplanned secondary objective, the second set of OA measurements were compared with the first set of OA measurements to assess intraobserver reproducibility using Bland–Altman plots. Intraobserver variation was analyzed by calculating the SD of the differences between the pairs of measurements made on each eye^29^. In addition, comparisons were made between the measurements in the left *vs* right eye.

Correlations between pairs of indices, and between Doppler indices and gestational age, were assessed using Pearson's or Spearman's rank correlation coefficient. A pairwise partial correlation analysis was undertaken using the R package ‘ppcor’ to adjust for the effect of maternal age and body mass index (BMI).

## RESULTS

**Figure 1:**
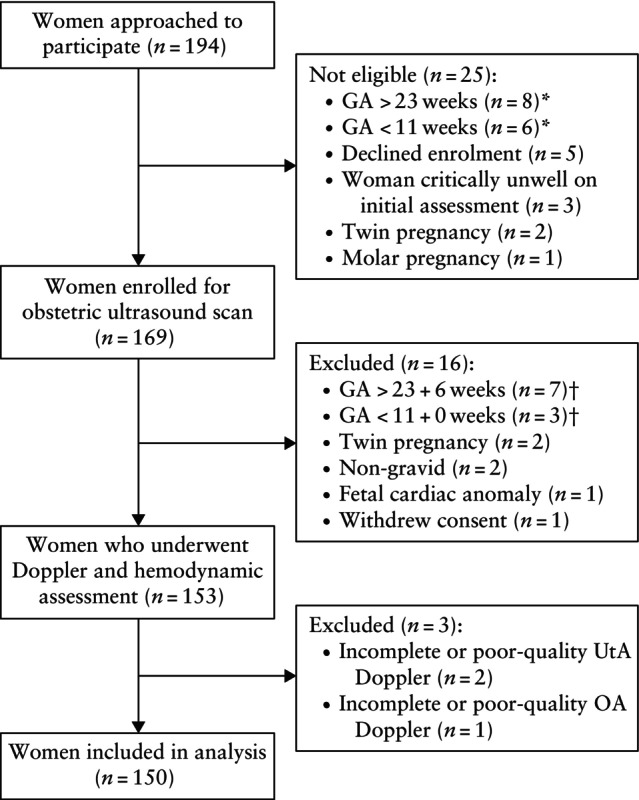
Flowchart summarizing inclusion of patients in study. *Based on last normal menstrual period, prior scan or obstetric abdominal examination. †Based on obstetric ultrasound scan performed as part of the study. GA, gestational age; OA, ophthalmic artery; UtA, uterine artery.

**Figure 2:**
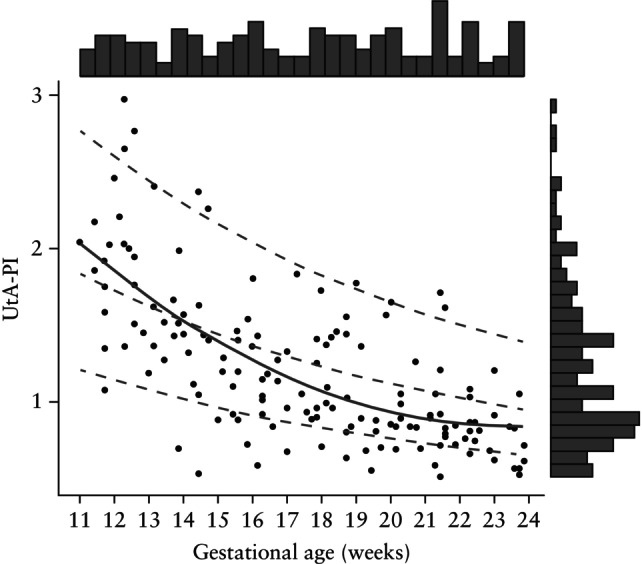
Relationship between gestational age (distribution shown horizontally) and uterine artery pulsatility index (UtA‐PI) (distribution shown vertically). Solid line indicates line of best fit in present (MATERA) cohort; dashed lines indicate 5^th^, 50^th^ and 95^th^ percentiles of reference range for UtA‐PI at 11–41 weeks' gestation by Gómez *et al*.^30^.

Overall, 194 women were invited to participate and 169 were eligible for participation. After exclusion of 16 participants following the obstetric ultrasound scan, and a further three owing to inadequate or incomplete UtA (*n* = 2) or OA (*n* = 1) Doppler assessment, 150 remained in the final analysis (Figure 1). As intended, the enrolment strategy ensured adequate representation of gestational ages between 11 + 0 and 23 + 6 weeks (Figure 2). Maternal and pregnancy characteristics of the participants are presented in Table 1. The mean ± SD maternal age was 26.5 ± 5.6 years, mean ± SD height was 155.8 ± 6.8 cm, median weight was 60.5 (IQR, 55.0–71.0) kg and median BMI was 25.5 (IQR, 22.3–29.6) kg/m^2^. Around one‐third (30.0%) of women were nulliparous and 31/112 (27.7%) had a history of pre‐eclampsia in a previous pregnancy.

**Table 1:** Maternal demographic and pregnancy characteristics of study population (*n* = 150)

Variable	Value
Maternal age (years)	26.5 ± 5.6
Maternal height (cm)*	155.8 ± 6.8
Maternal weight (kg)*	60.5 (55.0–71.0)
Maternal BMI (kg/m^2^)*	25.5 (22.3–29.6)
Education level	
Primary	34 (22.7)
Secondary	86 (57.3)
Tertiary/professional/technical	30 (20.0)
Systolic blood pressure (mmHg)*	123.8 ± 17.8
Diastolic blood pressure (mmHg)*	75.3 ± 12.1
Nulliparous	45 (30.0)
Previous pregnancy loss < 28 weeks	41/112 (36.6)
Previous pre‐eclampsia	31/112 (27.7)
Previous preterm birth	15/105 (14.3)
Previous birth weight < 2.5 kg	18/105 (17.1)
Previous birth weight (g)	3054.8 ± 783.8
Chronic hypertension	12 (8.0)
Human immunodeficiency virus	7 (4.7)
GA at presentation (weeks)	17.9 (14.4–20.8)

Data are given as mean ± SD, median (interquartile range), *n* (%) or *n*/*N* (%).

* Data missing for one patient.

BMI, body mass index; GA, gestational age.

### Correlation between Doppler indices and gestational age

**Figure 3:**
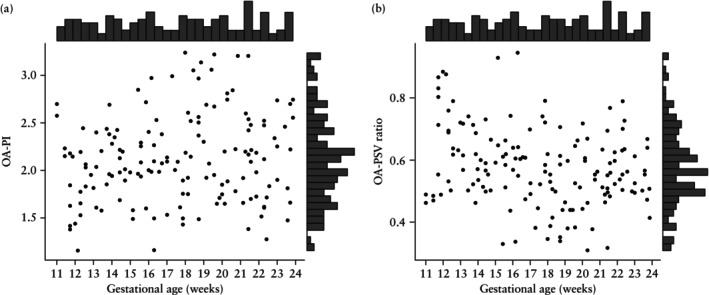
Relationship between gestational age (distribution shown horizontally) and ophthalmic artery (OA) Doppler indices (distribution shown vertically): (a) OA pulsatility index (PI); and (b) OA peak systolic velocity ratio (PSV ratio).

There was a strong and significant correlation between UtA‐PI and gestational age (*r* = –0.68 (95% CI, –0.76 to –0.58), *P* < 0.001) (Figure 2), which persisted after adjusting for maternal age (*r* = –0.68, *P* < 0.001) and maternal BMI (*r* = –0.68, *P* < 0.001). However, no significant correlation was seen between OA‐PI and gestational age (*r* = 0.14 (95% CI, –0.03 to 0.29), *P* = 0.097) (Figure 3a), and the correlation between OA‐PSV ratio and gestational age was weak (*r* = –0.23 (95% CI, –0.38 to –0.07), *P* = 0.004) (Figure 3b). These relationships remained unchanged after adjusting for maternal age (OA‐PI: *r* = 0.11, *P* = 0.180; PSV ratio: *r* = –0.20, *P* = 0.014) and maternal BMI (OA‐PI: *r* = 0.11, *P* = 0.181; PSV ratio: *r* = –0.21, *P* = 0.009).

### Correlation between UtA and OA Doppler indices

UtA‐PI was non‐normally distributed (Figure 2, Table 2). In contrast, OA indices showed no evidence of non‐normality (Figure 3, Table 2).

**Table 2:** Maternal Doppler indices and hemodynamic parameters in study population (*n* = 150)

Variable	Value
OA‐PI*	2.12 ± 0.46
OA‐PSV ratio*	0.58 ± 0.12
UtA‐PI*	1.08 (0.84–1.49)
Right OA‐PI	
First measurement	2.13 ± 0.53
Second measurement	2.10 ± 0.52
Left OA‐PI	
First measurement	2.11 ± 0.53
Second measurement	2.10 ± 0.54
Right OA‐PSV1	
First measurement	34.07 ± 10.39
Second measurement	34.59 ± 9.43
Right OA‐PSV2	
First measurement	19.54 ± 7.42
Second measurement	19.54 ± 7.42
Right OA‐PSV ratio	
First measurement	0.57 ± 0.14
Second measurement	0.62 ± 0.43
Left OA‐PSV1	
First measurement	34.31 ± 8.74
Second measurement	33.25 ± 8.39
Left OA‐PSV2	
First measurement	19.82 ± 6.44
Second measurement	19.11 ± 6.76
Left OA‐PSV ratio	
First measurement	0.58 ± 0.14
Second measurement	0.58 ± 0.15
Right UtA‐PI	
First measurement	1.01 (0.78–1.47)
Second measurement	1.03 (0.79–1.50)
Left UtA‐PI†	
First measurement	1.12 (0.83–1.56)
Second measurement	1.10 (0.84–1.54)
Mean arterial pressure (mmHg)†	91.47 ± 12.99
Systemic vascular resistance (dynes × s/cm^5^)†	987.57 ± 199.35
PE/KE ratio†	18.17 ± 4.78
Stroke volume (mL)†	93.39 ± 13.63
Cardiac output (L/min)†	7.65 ± 1.38
Heart rate (bpm)†	82.26 ± 10.95

Data are given as mean ± SD or median (interquartile range).

* Average of first set of right and left measurements.

† Data missing for one patient.

OA, ophthalmic artery; PE/KE ratio, potential energy/kinetic energy ratio; PI, pulsatility index; PSV ratio, peak systolic velocity ratio (PSV2/PSV1); PSV1, first peak systolic velocity; PSV2, second peak systolic velocity; UtA, uterine artery.

To assess the primary outcome, UtA‐PI was log‐transformed to normalize its skewed distribution (Figure S1). There was a significant but weak correlation between log UtA‐PI and OA‐PI (*r* = –0.19 (95% CI, –0.34 to –0.03), *P* = 0.021). The relationship was similar after adjusting for maternal age (*r* = –0.19, *P* = 0.022) and maternal BMI (*r* = –0.17, *P* = 0.041). Similarly, the correlation between log UtA‐PI and OA‐PSV ratio was significant but weak (*r* = 0.31 (95% CI, 0.15–0.45), *P* < 0.001), and remained unchanged after adjusting for maternal age (*r* = 0.31, *P* < 0.001) and maternal BMI (*r* = 0.30, *P* < 0.001).

### Correlation between OA Doppler and maternal hemodynamic indices

Maternal hemodynamic parameters were transformed into *Z*‐scores before conducting a correlation analysis. No significant correlations were observed between OA‐PI and maternal hemodynamic indices, with the exception of PE/KE ratio *Z*‐score (*r* = –0.17 (95% CI, –0.32 to –0.01), *P* = 0.041) (Figure S2, Table 3). This correlation remained significant after adjusting for maternal BMI (*r* = –0.18, *P* = 0.027), and showed a trend towards significance after adjusting for maternal age (*r* = –0.16, *P* = 0.078).

**Table 3:** Correlation between ophthalmic artery Doppler indices and maternal hemodynamic indices

Variable	*r* (95% CI)	*P*
Ophthalmic artery PI *vs*:		
MAP *Z*‐score	–0.09 (–0.24 to 0.08)	0.299
Heart rate *Z*‐score	–0.04 (–0.20 to 0.12)	0.595
SVR *Z*‐score	–0.08 (–0.24 to 0.08)	0.313
Cardiac output *Z*‐score	–0.04 (–0.20 to 0.12)	0.650
Stroke volume *Z*‐score	0.01 (–0.16 to 0.17)	0.954
PE/KE ratio *Z*‐score	–0.17 (–0.32 to –0.01)	0.041
Ophthalmic artery PSV ratio *vs*:		
MAP *Z*‐score	0.29 (0.13 to 0.43)	< 0.001
Heart rate *Z*‐score	–0.08 (–0.24 to 0.08)	0.333
SVR *Z*‐score	0.23 (0.07 to 0.37)	0.005
Cardiac output *Z*‐score	0.02 (–0.14 to 0.18)	0.811
Stroke volume *Z*‐score	0.08 (–0.08 to 0.24)	0.332
PE/KE ratio *Z*‐score	0.26 (0.11 to 0.41)	0.001

MAP, mean arterial pressure; PE/KE ratio, potential energy/kinetic energy ratio; PI, pulsatility index; PSV, peak systolic velocity; SVR, systemic vascular resistance.

OA‐PSV ratio was found to be correlated weakly but significantly with MAP *Z*‐score (*r* = 0.29 (95% CI, 0.13–0.43), *P* < 0.001), SVR *Z*‐score (*r* = 0.23 (95% CI, 0.07–0.37), *P* = 0.005) and PE/KE ratio *Z*‐score (*r* = 0.26 (95% CI, 0.11–0.41), *P* = 0.001). These correlations persisted after adjusting for maternal age (*r* = 0.22, *P* = 0.006; *r* = 0.22, *P* = 0.007; and *r* = 0.22, *P* = 0.006, respectively) and maternal BMI (*r* = 0.26, *P* = 0.001; *r* = 0.22, *P* = 0.006; and *r* = 0.28, *P* < 0.001, respectively).

### Reproducibility of OA Doppler indices

**Figure 4:**
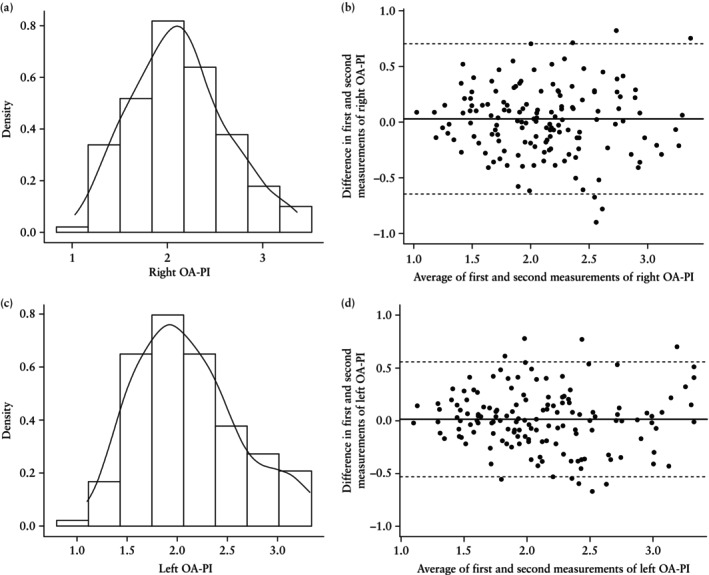
Distribution of ophthalmic artery (OA) pulsatility index (PI) (a,c) and Bland–Altman plots for intraobserver reproducibility of OA‐PI measurement (b,d) for the right eye (a,b) and left eye (c,d). Solid line in (b,d) is mean difference and dashed lines are 95% limits of agreement.

**Figure 5:**
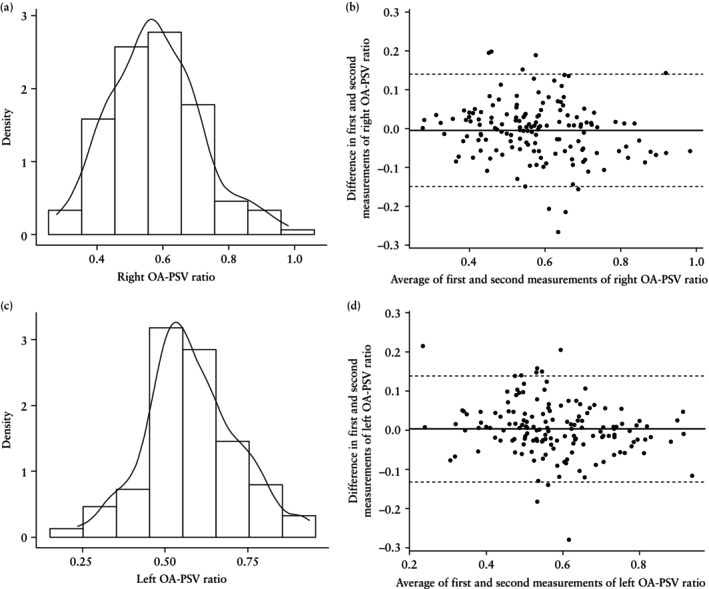
Distribution of ophthalmic artery (OA) peak systolic velocity ratio (PSV ratio) (a,c) and Bland–Altman plots for intraobserver reproducibility of OA‐PSV ratio measurement (b,d) for the right eye (a,b) and left eye (c,d). Solid line in (b,d) is mean difference and dashed lines are 95% limits of agreement.

OA‐PI and OA‐PSV ratio were measured twice in each eye in all 150 women. The distributions of the mean OA‐PI for the right eye and left eye, as well as Bland–Altman plots for intraobserver reproducibility, are shown in Figure 4. The respective data for OA‐PSV ratio are shown in Figure 5. There was no significant correlation between OA‐PI or OA‐PSV ratio and measurement error, meaning that reproducibility could be expressed using the SD of the difference.

For OA‐PI, the mean ± SD of the difference between the first and second measurements was 0.028 ± 0.345 and 0.013 ± 0.281 for the right and left eye, respectively. On 95% of occasions, the measurement of the right OA‐PI did not differ by more than ± 0.675, while for the left eye, OA‐PI did not differ by more than ± 0.550. For OA‐PSV ratio, the mean ± SD of the difference between the first and second measurements was –0.004 ± 0.074 and 0.0037 ± 0.0686 for the right and left eye, respectively. On 95% of occasions, the measurement of the right OA‐PSV ratio did not differ by more than ± 0.145, while for the left eye, OA‐PSV ratio did not differ by more than ± 0.135.

### OA Doppler indices in right *vs* left eye

Measurements of OA‐PI in the right and left eyes were moderately but significantly correlated (*r* = 0.63 (95% CI, 0.53–0.72), *P* < 0.001) (Figure S3a). For OA‐PSV ratio, the correlation between measurements in the right and left eyes was strong (*r* = 0.81 (95% CI, 0.75–0.86), *P* < 0.001) (Figure S3b).

## DISCUSSION

The principal finding of this study was the lack of a strong correlation between UtA‐PI and either OA‐PI or OA‐PSV ratio. We also found that: (1) gestational age had a weak effect on OA‐PSV ratio and no effect on OA‐PI, meaning that these indices could be measured without adjustment at any timepoint between 11 + 0 and 23 + 6 weeks' gestation, which is important for settings in which early antenatal care is frequently lacking; (2) OA Doppler indices were highly reproducible; (3) OA Doppler indices were correlated strongly between the right and left eyes; and (4) there was a significant but weak correlation between OA‐PI and PE/KE ratio *Z*‐score, and between OA‐PSV ratio and *Z*‐scores of MAP, SVR and PE/KE ratio. Although the correlations were weak, some of the relationships may be of clinical significance.

We demonstrated a strong correlation between UtA‐PI and gestational age (*P* < 0.001), as expected from previous work^30^. In contrast, no‐to‐little correlation was seen between OA‐PI or OA‐PSV ratio and gestational age at 11 + 0 to 23 + 6 weeks of gestation. While the study of Carneiro *et al*.^18^ demonstrated no correlation between OA Doppler indices and gestational age at 20–39 weeks, Kumari *et al*.^17^ found contradictory results in a cross‐sectional study of 40 hypertensive and 40 normotensive pregnant women, in which they reported a negative correlation between OA‐PI and gestational age. de Oliveira *et al*.^16^ also reported a weak but significant correlation at 20–40 weeks.

In the present study, we found weak correlation between OA‐PI or OA‐PSV ratio and log UtA‐PI. A published review suggested that OA‐PSV ratio has significant complementary predictive value to UtA‐PI in pre‐eclampsia^8^. The OA‐PSV ratio also had important standalone predictive value for subsequent pre‐eclampsia among pregnancies at 19–23 weeks^9^. Although we do not intend to recommend exclusion or replacement of UtA Doppler with OA Doppler in pre‐eclampsia prediction models, OA Doppler indices have several advantages, such as invariable reference values throughout pregnancy and the fact that they are unaffected by adiposity^31^. Finally, OA Doppler could be of particular interest in LMIC settings. Although a relatively specialized application, OA Doppler is thought to be easier to learn than UtA Doppler because the OA is relatively easy to access via transorbital ultrasound and the anatomy is less complex, as there is a single predominant vessel unlike the more variably located UtA. In addition, since the OA is close to the probe on transorbital Doppler imaging, the images obtained are clear and easy to interpret, and this may accelerate the learning curve.

Consistent with prior research, we found weak but statistically significant correlations between OA‐PSV ratio and several maternal hemodynamic indices. In a prospective study at 19–23 weeks, Gibbone *et al*.^32^ found a significant correlation between OA‐PSV ratio and left ventricular mass in pre‐eclamptic women, a phenomenon thought to be a secondary adaptation to increased SVR. However, no such relationship was observed among normotensive women^32^. Lau *et al*.^12^ observed a significant association between MAP and OA‐PSV ratio (*P* < 0.001), which is consistent with the finding reported by Sapantzoglou *et al*.^9^ (*r* = 0.15 (95% CI, 0.11–0.19)). The correlation coefficient between OA‐PSV ratio and MAP *Z*‐score in our study was 0.29, almost double that reported previously in high‐income settings^9^, ^32^. This could perhaps be attributed to a greater proportion of higher‐risk participants in our study, as well as a greater proportion of women at < 20 weeks' gestation compared with many previous studies. Increases in MAP in hypertensive disorders of pregnancy primarily lead to hypoperfusion and end‐organ injury. In the brain, hypoperfusion damages the blood–brain barrier and distorts cerebral autoregulation. Distorted autoregulation has been associated with an increase in cerebral blood flow, including in the OA^17^.

Both OA‐PI and OA‐PSV ratio were characterized by a high degree of intraobserver reproducibility within the same eye. It is imperative for a test to be reproducible for clinical application, and our findings are consistent with previous studies in women after 20 weeks^16^, ^18^. In addition, no significant difference between OA‐PI measurements in the right *vs* left eye was seen. This consistency can be enhanced by ensuring that measurements are taken at the same point along the OA^16^.

Strengths of our study include the examination of OA Doppler in the context of pre‐eclampsia screening in sub‐Saharan Africa over a relevant gestational‐age range. We used a stratified and random sampling approach to ensure that we included at least 10 participants per gestational week from 11 to 23 weeks. We included both low‐ and high‐risk individuals, enhancing generalizability. OA and UtA Doppler indices were measured in duplicate by the same operator and were obtained in accordance with recommended methodology regarding anatomical features, magnification and angle of insonation. Our study provides further evidence that OA Doppler represents a non‐invasive, reproducible and easily applicable complementary screening technique that may be of relevance to LMICs.

One limitation of our study was the relatively small sample size, which precluded determination of the screening performance of OA Doppler for pre‐eclampsia, but this was beyond the scope of this work. In our view, to ascertain whether OA Doppler is useful for pre‐eclampsia screening in LMIC settings, it will be essential to first test the correlation between OA Doppler and other screening tests, and second to establish the reproducibility of the method. Only then would it be reasonable to embark on large‐scale studies to assess screening characteristics, which we are now planning.

In conclusion, OA‐PI and OA‐PSV ratio had a weak or no correlation with UtA‐PI and maternal hemodynamic parameters, meaning that they can be used as independent predictors of pre‐eclampsia at 11 + 0 to 23 + 6 weeks' gestation. Gestational age had no clinically relevant effect on OA‐PI or OA‐PSV ratio, meaning that these indices could be measured without adjustment at any time over this gestational‐age range. Implementation of OA Doppler appears to be feasible and scalable, with a high degree of intraobserver reproducibility. OA Doppler indices correlated strongly between the right and left eyes. These findings suggest that OA Doppler may be a useful point‐of‐care clinical test for predicting complications relating to hypertensive disease in pregnancy and warrant further validation in a large‐scale study.

## Supporting information


**Figure S1** Histograms showing distribution of uterine artery pulsatility index (PI) before and after log transformation.
**Figure S2** Relationship between ophthalmic artery Doppler indices (peak systolic velocity ratio (PSV ratio) in (a–f) and pulsatility index (PI) in (g–l)) and selected maternal hemodynamic indices. CO, cardiac output; HR, heart rate; MAP, mean arterial pressure; PKR, potential energy/kinetic energy ratio; SV, stroke volume; SVR, systemic vascular resistance.
**Figure S3** Relationship between ophthalmic artery Doppler measurements in left and right eyes: (a) pulsatility index (PI); and (b) peak systolic velocity ratio (PSV ratio).

## Data Availability

The data that support the findings of this study are available from the corresponding author upon reasonable request.
