# Supplementary material for: Relationship of maternal ophthalmic artery Doppler with uterine artery Doppler, hemodynamic indices and gestational age: prospective MATERA study
Source: Ultrasound Obstet Gynecol. 2025 Jan 20;65(2):163–72. doi: 10.1002/uog.29162 (PMC11788460; doi:10.1002/uog.29162)
Supplement: Supplementary file 1 — Figure S1 Histograms showing distribution of uterine artery pulsatility index (PI) before and after log transformation. Figure S2 Relationship between ophthalmic artery Doppler indices (peak systolic velocity ratio (PSV ratio) in (a–f) and pulsatility index (PI) in (g–l)) and selected maternal hemodynamic indices. CO, cardiac output; HR, heart rate; MAP, mean arterial pressure; PKR, potential energy/kinetic energy ratio; SV, stroke volume; SVR, systemic vascular resistance. Figure S3 Relationship between ophthalmic artery Doppler measurements in left and right eyes: (a) pulsatility index (PI); and (b) peak systolic velocity ratio (PSV ratio). [file UOG-65-163-s001.docx]

**Supplementary material**

Figure S1 Histograms showing distribution of uterine artery pulsatility index (PI) before and after log transformation.


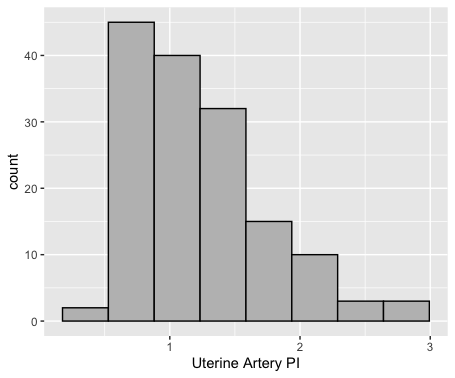

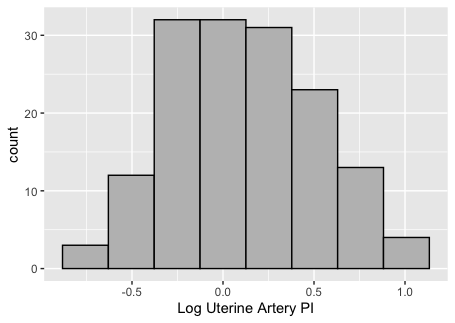


**Figure S2** Relationship between ophthalmic artery Doppler indices (peak systolic velocity ratio (PSV ratio) in (a–f) and pulsatility index (PI) in (g–l)) and selected maternal hemodynamic indices. MAP, mean arterial pressure; HR, heart rate; SVR, systemic vascular resistance; CO, cardiac output; SV, stroke volume; PKR, potential energy/kinetic energy ratio.

| 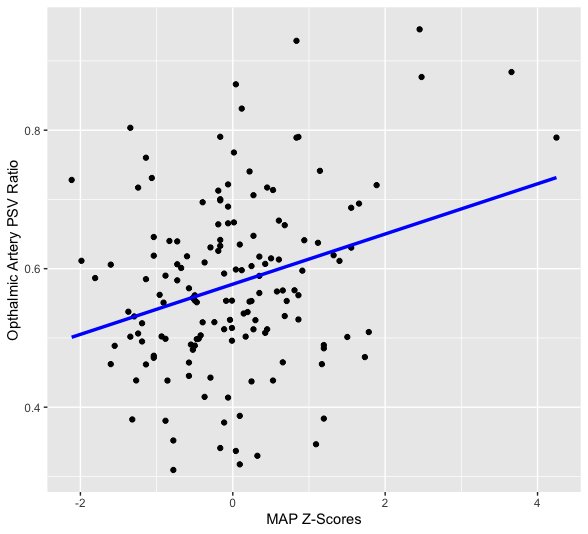^a)^ | 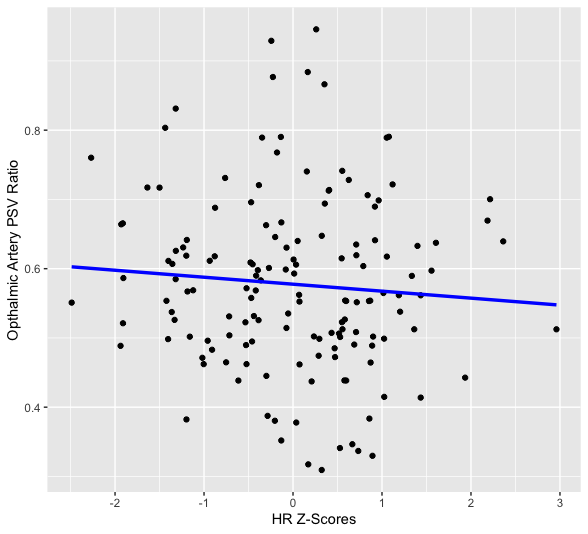^b)^ |
| --- | --- |
| 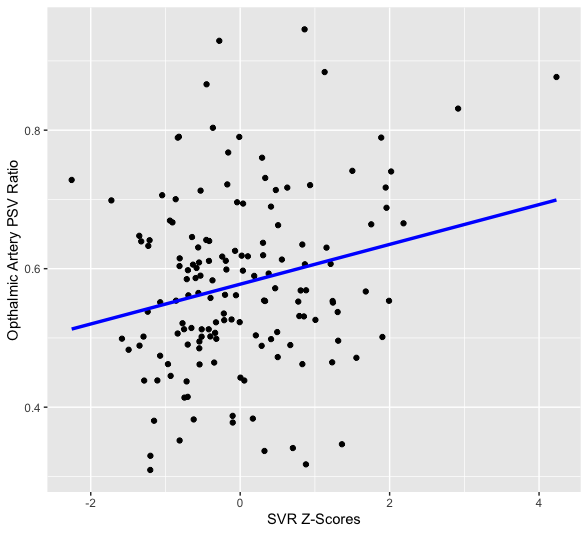  ^C)^ | 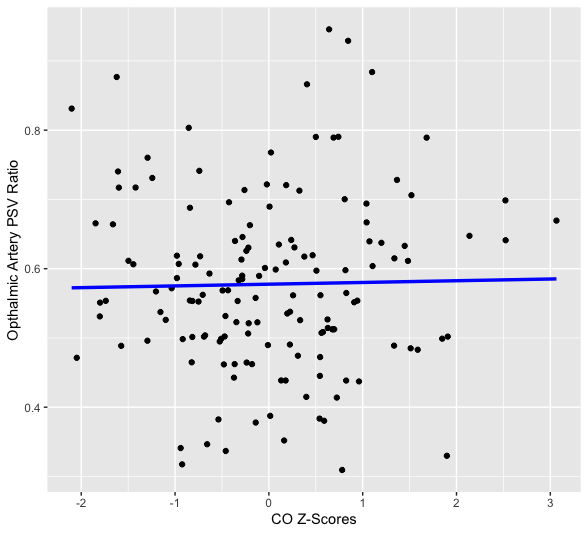  ^d)^ |
| 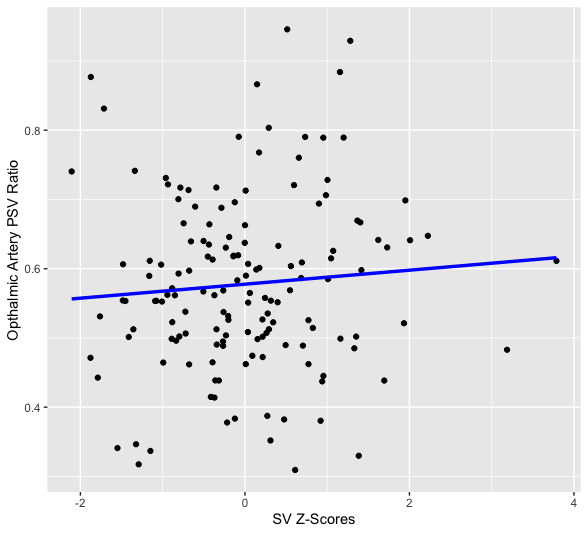  ^e)^ | 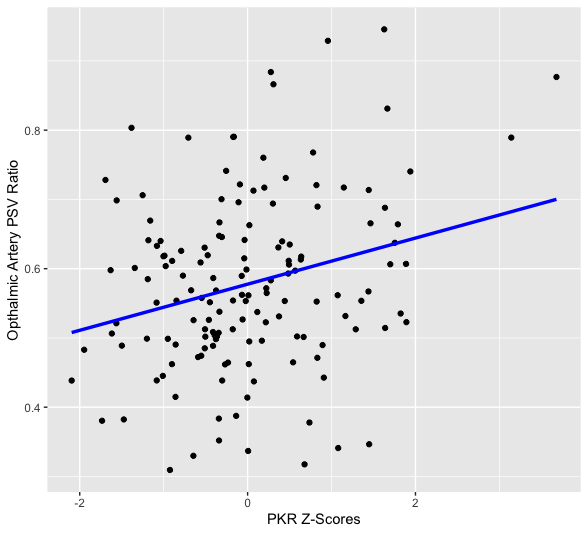  ^f)^ |
| 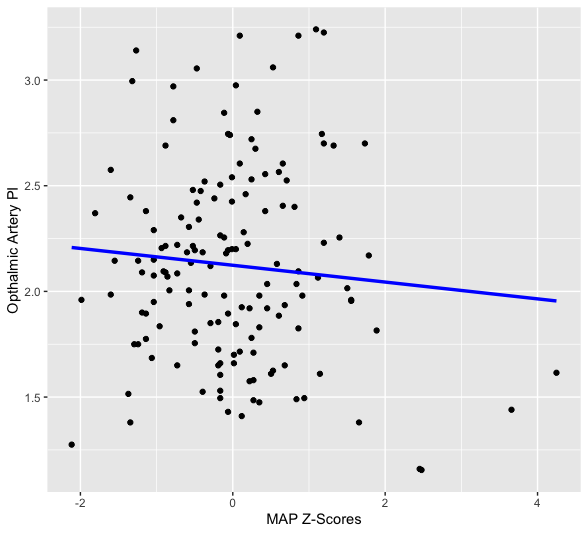  ^g)^ | 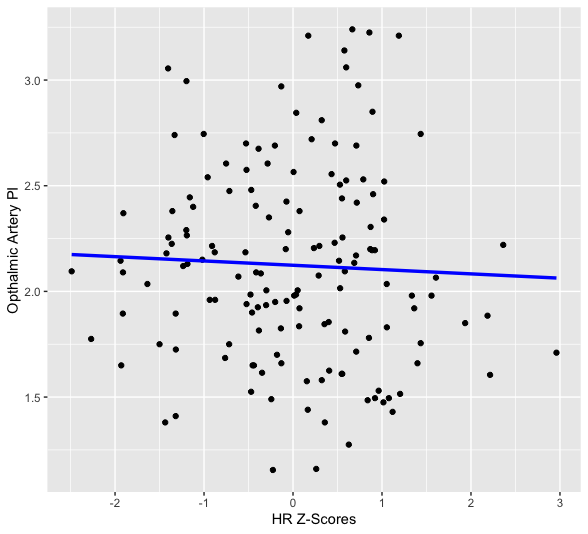  ^h)^ |
| 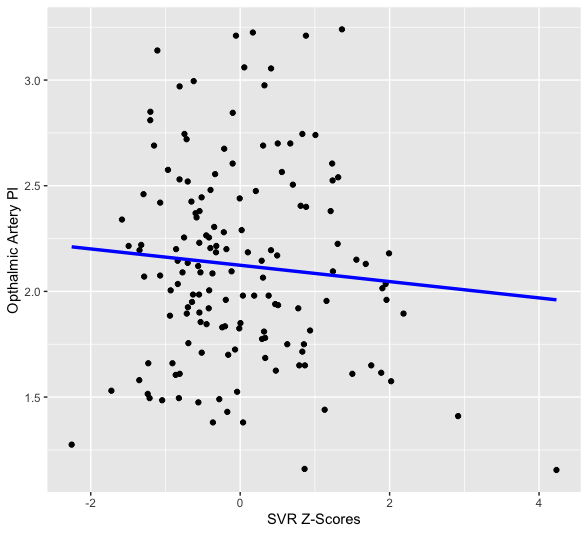  ^i)^ | 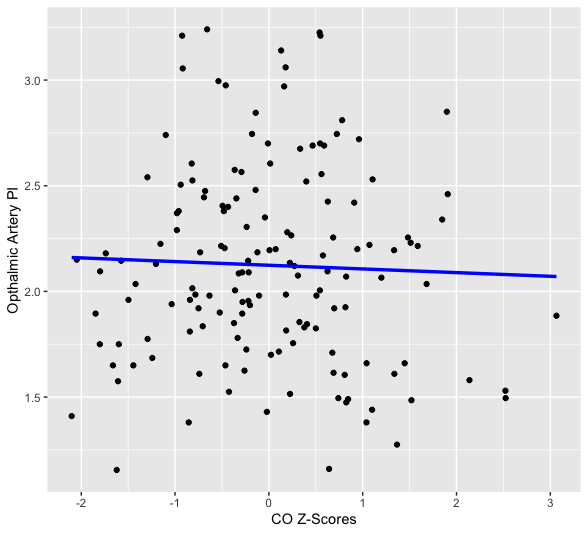  ^j)^ |
| 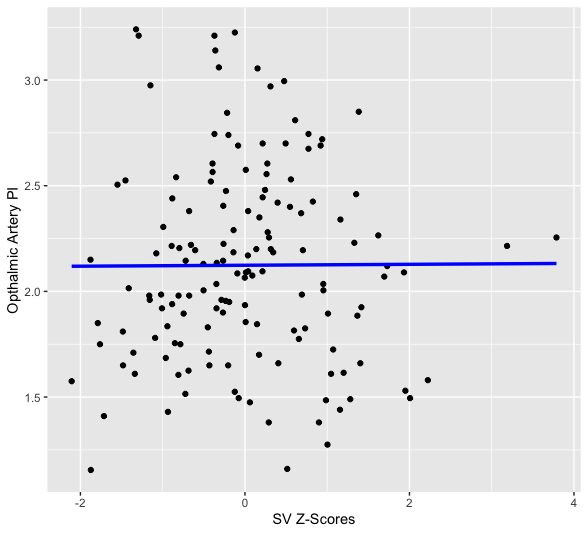  ^k)^ | 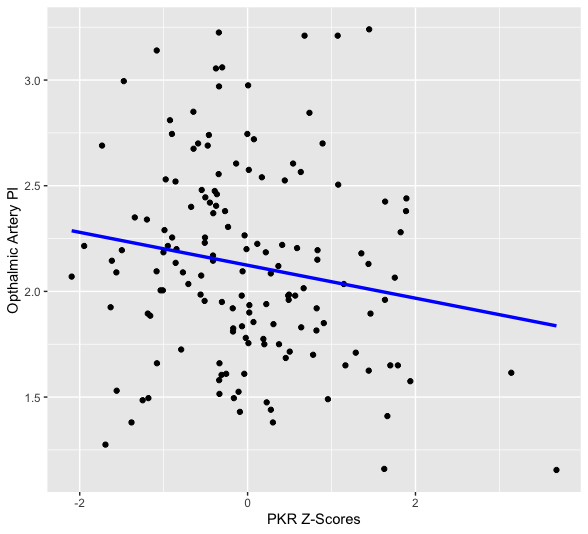  ^l)^ |

**Figure S3** Relationship between ophthalmic artery Doppler measurements in left and right eyes: (a) pulsatility index (PI); and (b) peak systolic velocity ratio (PSV ratio).

| 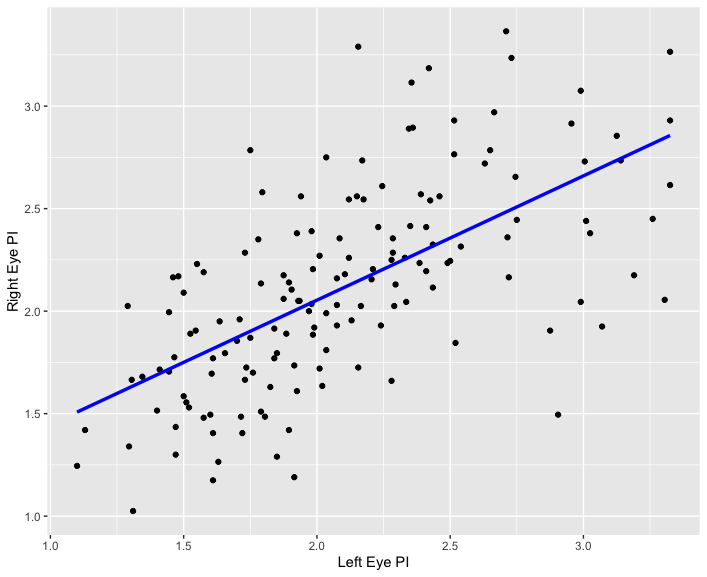  a) | 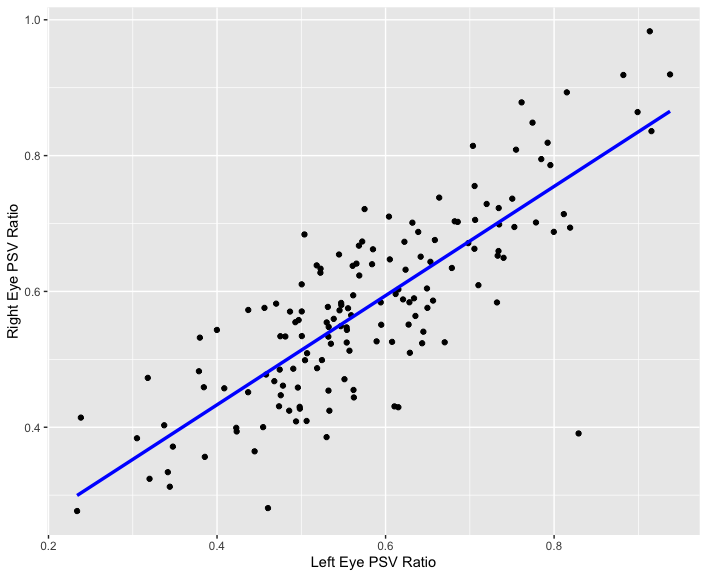  b) |
| --- | --- |
